# Dr. Elliotson on Glanders in the Human Subject

**Published:** 1831-01-01

**Authors:** 


### Glanders in the Human Subject. By *Dr. Elliotson.*

[Medico-Chirurgical Transactions.]

This is a very extended paper, occupying 35 pages, and will require a minute analysis.

*Case* 1. Thomas Maskall, aged 17 years, was admitted into St. Thomas's Hospital, under Dr. Roots, on the 19th March, 1829. The upper part of the face, including the eyelids and the nose, with portions of the cheek on each side, were greatly tumefied, so that the eyes were closed. The eyelids and swollen portions of the cheeks were red and hot, dry and shining; but the nose was dark-coloured, and one side of it was black, cold, and senseless. From around the gangrened portion of the nose and some other parts, a little pus was oozing, as well as a thin and dark-coloured fluid, and from *the nostrils a thick discharge of a deep yellow colour, here and there a little, was taking place, exceedingly copious from the right nostril.* Several hard phlyzacious pustules existed around the nose, and on various parts of the trunk and members. Tumefactions were observed on both fore-arms, and in the back of the right hand. The pulse was 136, the skin hot, tongue white and rather dry, respiration quick and difficult. Bleeding was employed—the blood was buffed—some of the tumefactions were opened and discharged much pus. Tumefactions now appeared on various other parts of the body—the first ones had acquired a purple tint, and the wounds looked dark. There was constant agitation, delirium, profuse diarrhœa, insomnolency. He gradually sunk, and died the next day.

"I was not present at the inspection, but understand that there were proofs of a violent pleuro-peripneumonia in the lower part of the right side, and that two ounces of healthy pus were found between the adherent lung and diaphragm. A dark red patch existed in the stomach, and several at irregular distances throughout the intestines. The mucous membrane of the stomach was very lacerable at that spot. The other viscera, and all the contents of the head as well as the veins of the extremities were reported healthy: but the mucous membrane of the nostrils was not examined. The abscess which had been opened in the back of the right hand communicated with the joint of the metacarpal bone of the middle finger, but those on the arm did not communicate with the elbow." 175.

This case was perfectly new to the medical officers of the hospital. It was designated "gangrena nasi," and other appellations were bestowed on it, but not one could elucidate its nature.

On Friday, the 26th of the following June, Dr. Elliotson was surprised to find another young man, with similar symptoms, lying in William's ward. The symptoms we need not repeat. It appeared that he worked with his father, a blacksmith—that two months previously he had taken a large draught of cold porter when hot and perspiring, and had not afterwards felt well. Three weeks prior to entrance into the hospital, he had been attacked with acute rheumatism, from which he was recovering, when a pimple appeared on his nose, succeeded by a blush of inflammation that grew rapidly more intense, when he was sent to the hospital, and ordered colchicum by the apothecary. He had never had any venereal affection or taken mercury, to his knowledge. Dr. E. ordered 50 minims of laudanum and 10 grains of sulphas quinæ to be given immediately, and the latter to be repeated every hour.

"In two hours I visited him again, but found the disease had made great progress in overpowering the system. The elbow, which had been red only before, was now suppurating: the pulse was 166, still smaller and fluttering, and the restlessness was extreme. I instantly gave him ♏. l. more of the tinctura opii, and directing the sulphas quininæ to be continued, and as much strong beef-tea, with eggs diffused in it to be supplied as he would take. Neither the nourishment, however, nor any more of the sulphas quininæ, could he be prevailed upon to taste: but he fell asleep soon after this second dose of laudanum, and slept tranquilly, waking occasionally for a minute and turning on the other side, till four o'clock in the morning, when he became very restless for an hour and then expired." 180.

As in the former case, the father was present at the inspection, and would not allow the head to be opened. Many parts of the lungs were gorged with blood and frothy fluid, the corresponding bronchial branches being very dark. There were marks of irritation, or even inflammatory action in the glands of the small intestines. The parietes of the left ventricle were in a state of hypertrophy. Nothing, however, in the dissection was calculated to throw any light on the true nature of the disease, which was awful from its suddenness, rapidity, and gangrenous symptoms. Comparing it with the preceding case, our author came to the opinion of their identity, and concluded that they were specific eruptive fevers—that they were caused by some morbid poison—which poison was furnished by a living system under a similar disease. Still the nature and source of the poison remained a mystery, till Dr. E. saw on the cover of the Medical Gazette, July 4th, "a fatal case of *acute* glanders in the human subject," when it instantly flashed across his mind, that this was the disease by which he had been puzzled. Here follows the case from the Gazette, of which we shall take but a very brief notice.

A corporal in the 2d Regiment of Dragoons, awoke with rigors, headache, and irritability of the stomach, succeeded by depression of spirits, pains in his joints, tumefaction of the left shoulder, &c. In eight days this tumefaction was livid, and similar swellings took place on the arms, legs, thighs, and sacrum, cracking and exuding a thin acrid sanies. "The right nostril was gummed with an inspissated discharge." The posterior fauces were much inflamed, and nearly of a purple hue. In four days more, several "warty pustules," the size of a pea, arose high above the skin in various parts of the body, together with a host of anomalous and varying phenomena, which it would be endless as well as needless to describe. By the 12th day many of the tumours were running into gangrene—the pulse was scarcely perceptible, and on the 14th day he died.

"Neither the thoracic nor abdominal viscera presented any vestige of disease, but, as there was in this case no impediment to a full examination, the head, extremities, and walls of the trunk were carefully inspected. A cluster of tubercles was found in the cellular membrane, exterior to the pericranium of the left superciliary ridge, and in the right frontal sinus, exactly, according to the veterinary surgeon of the regiment, similar to those observed in the frontal and other sinuses of the horse after acute glanders. On dividing the various livid tumours of the surface down to the bone, 'the muscles appeared perfectly decomposed, and of a dark liver colour, exhaling a peculiarly fetid odour, with points of purulent matter, as it were infiltrated every where through its entire substance, resembling much a hepatized or tuberculated lung;' and under each 'was a cluster of grey circular tubercles, the whole composed of fine cellular tissues, enclosed in small cysts, proportionate in size and consistency to the extent and duration of the tumour, and firmly attached to the periosteum.' The muscles generally, even perhaps the heart, appeared pale and flabby, 'and the cellular membrane infiltrated with a yellow serosity.'

'It appeared that the patient had had the sole charge of a glandered horse for some time, which had been destroyed on the very evening of his attack; and that he had skinned him, and exerted himself a good deal in cutting up and burying the carcass. But these circumstances did not at first create the least suspicion, and his complaint was considered a very severe case of acute rheumatism, and treated as such.'" 185.

If the two cases which occurred at St. Thomas's Hospital tallied with each other, they did not less perfectly correspond with the foregoing case, and our author felt a conviction that an active investigation would discover an identity of cause. He therefore repaired to the house of the father of his own patient, in Lambeth. The forge was situated in a mews; and it was ascertained that a glandered horse had been kept for six weeks in a shed separated from the place where the young man had worked, only by a thin partition, so defective that the discharge from the animal's nostrils came through the boards, and occasioned an unbearable stench. When the animal was leading to the knacker's, about a month before the commencement of his rheumatism, he fell down opposite to the forge, and the young man went out and patted him about the head. It appeared that he had a habit of wiping his eyes with the back of his hand, and for some time had been troubled with pimples on his forehead and about the nose. Encouraged by this denouement, Dr. E. proceeded to inquiries respecting the other individual, Maskall. He had come from Woolwich, and to that place Dr. E. repaired in July, but the search for Maskall was in vain. Subsequently, however, it was ascertained that a next-door neighbour of the lad kept a miserable poney covered with sores and lodged in a wretched shed. In the cart which this animal drew, the young man often got a ride, and he frequently helped to harness and unharness him. It was sent to the knackers a few months after the boy's death. It was subsequently ascertained that this poney actually had the glanders. The father of the boy also remembered that he had been troubled sometime before his illness with pimples on his forehead and chin, and our author thinks it highly probable that some of the glandered matter came in contact with these eruptions, when they happened to be open.

Meeting Mr. Parrott, of Clapham, one day, Dr. E. mentioned to him these two cases, and his conviction that they were instances of glanders. Soon afterwards, Mr. P. furnished our author with the particulars of the following case.

"Mr. John Vass, aged 23, a pupil of the Veterinary College, had under his care at Clapham, where his father resided, a horse affected, the father said, with 'farcy glanders.' The ring-finger of the right hand, and the absorbents and axillary glands, became inflamed and painful; but whether after any wound or abrasion could not be satisfactorily ascertained. The finger suppurated, and was opened; the aperture healed quickly, all the inflammation subsided, and in a few days he was considered free from complaint, except that his temper was irritable and his appetite somewhat impaired. But in a few days more he began to feel headache and pains in his limbs, particularly in the right-knee. On the following day, (March 27) Mr. Parrott first saw him, and could detect no tenderness, swelling, hardness, nor trace of previous wound in the extremity originally affected. The pain in the knee was very severe, and aggravated by motion, but unattended by heat, swelling or redness. The pulse was quick and full; the tongue white and moist; the skin hot, and bedewed with sweat; the urine scanty, high-coloured, and turbid; the countenance anxious; the head painful, and there were signs of cerebral disturbance, together with extreme restlessness; the throat was sore, and covered with aphthous specks; the palpebræ were swollen, and the eyes much suffused. All these symptoms continued with unabated violence for eight or nine days; and in the mean time, numerous soft swellings took place in the extremities, the small joints became painful and rather red, the right knee enormously swollen, the absorbents of the right arm knotted, painful, and in many points distinctly fluctuating. *A copious sero-mucous discharge, occasionally a little bloody, occurred from the eyes and nose, the schneiderian membrane was excessively red and nearly excoriated, and the eyes closed.* A pretty abundant eruption, very similar to small-pox, but larger, and *hard,* appeared in different parts, but particularly in the neck. There was scarcely any sleep, but occasional delirium, and at length convulsions, and the patient expired on the 7th of April, while changing his linen. Unhealthy pus was found in the absorbents of the arm; the bursa of the knee contained a large quantity of pus, with flakes of coagulable lymph, a considerable abscess existed on the inner side of the knee-joint, and the periosteum was detached to the extent of between three or four inches. No other parts were examined." 192.

Dr. E. here introduces a description of acute glanders, as seen in the horse and ass, from a veterinary writer in the Dict. de Med. et Chir. Veter.; but this we need not quote. Sir Gilbert Blane has stated, that "the only examples hitherto ascertained" of infection being communicated from one species to another, are "the hydrophobia and cow-pox"—and Mr. Coleman declares that, as far as his experience goes, "the nostrils of the human subject are not susceptible of glandered ulceration or inflammation."

"On applying, however, at the Veterinary College, I was informed by Mr. Sewel that he had known *two* instances, and referred me to Mr. Travers's book on Constitutional Irritation for the particulars. They were cases of *chronic* glanders. The *first* happened in a veterinary student, who slightly injured his hand in examining the head of an ass, which had died of inoculated glanders. An ulcer ensued, and pain and inflammation of the superficial absorbents took place in a few days, and soon ceased. But the absorbents of the opposite arm became affected, and an abscess formed in it, and another at the lower part of the back. He became hectic, and at length suppuration occurred also in the lungs, in one of the kidneys, and successively in each knee-joint; after which he died." 195.

Mr. Colman inoculated an ass over the maxillary gland, with matter from an abscess in the arm, and likewise rubbed the schneiderian membrane. Glanders and farcy were the result, and the animal died on the twelfth day. Precisely the same was done with another ass; but no effect ensued, as the matter was not employed for several days, and had been left exposed to the air. He repeated the same experiment upon the same animal with fresh matter, and it perished of glanders and farcy upon the 14th day.

Dr. E. introduces quotations from veterinary writers to shew that glanders and farcy are the same disease, merely affecting different parts. Farcy always appears to terminate ultimately in glanders. A case is quoted from Mr. Travers' work on Constitutional Irritation, where the disease commenced in the form of farcy, and at length produced the symptoms of glanders. In order to make this article as complete as possible, we shall introduce this case here.

"Nimrod Lambert, a healthy hackney coachman, æt. 32, in January, 1822, infected a chap on the inside of the right thumb, by inserting it into the nostril of a glandered horse, to pull off a scab. He remembered to have afterwards wiped the thumb with a wisp of hay. In the space of six hours, he was seized with violent pain and swelling of the thumb; it inflamed rapidly, upon which he applied a poultice to it, and took some salts. On the third day he was suddenly taken ill whilst driving, with cold shivers and giddiness, and states that he entirely lost the use of his limbs for seven hours. At this time his arm pained him much all the way up, and on the following day it was streaked with red lines, and excessively swollen. The arm-pit was also much swollen and tender. In the evening of the fourth day he was carried to Guy's Hospital, where he lay during twenty-four weeks. Superficial collections of matter formed successively in the course of the absorbents. The corresponding portions of the integument sloughed, leaving extensive ulcers which discharged an unhealthy and fetid matter. The glands at either angle of the lower jaw, and those of the groin became swollen, and he was much afflicted with pain between the eyes and down the nose, and exulcerations of the membrana narium, attended with discharge. During the progress of the local disease he had much constitutional illness. He totally lost his appetite, and was oppressed with nausea; complained of severe pains, with swimming in the head, and occasionally wandered in mind. He had also much pain through the whole course of the spine, especially in the region of the kidneys. His urine was thick, and discoloured, and fetid; his motions were slimy and purulent. Expecting to die, he quitted the hospital, and lay at home the remainder of the twelvemonth, in a state of great emaciation, from the continued discharge of his sores, his inability to take food, and to procure any refreshing sleep, even with the assistance of opiates, which he took habitually. Despairing of aid from the profession, he applied to an experienced female practitioner, who administered a decoction of herbs, which he invariably vomited, but to which nevertheless he ascribed his recovery. At the end of the twelvemonth, his health gradually returned, the arm began to heal, and he became comparatively hearty, and resumed his occupation, though with much inconvenience, owing to the distortion of his hand by the retraction of the thumb and forefinger, in the cicatrization of a long line of abscesses, reaching to the middle of his upper arm. After six weeks, this cicatrix ulcerated afresh, and healed slowly. He is still subject to wandering pains in the head, both sides of the neck, loins, and groins: is not so strong and so fleshy as formerly, but has a good appetite. He has a great heaviness and disposition to sleep during the day, and at the end of two years and a half from the breaking out of the disease, considers his constitution broken, and despairs of being ever again the man he was." 201.

As two if not three cases of this disease are now recorded with Dr. E.'s own observation, and another in another place, our author thinks it not unreasonable to conclude that the disease is not of extreme rarity. This supposition will be the more readily assented to, when the following cases, recorded in Rust's Magazine of the Healing Art, are perused. They were furnished to Dr. E. by Dr. Kind, a German physician residing in London, and Mr. Jacob, an excellent German scholar, both of whom had witnessed the cases in St. Thomas's Hospital. We shall give these curious and interesting cases a place in our Journal.

"I. Martin Rennspiess, aged 34, an artillery soldier, employed particularly in the care of horses at a veterinary school, felt unwell, and complained of rheumatism, catarrh, colic pains and thirst, but did not lie by. At the end of six weeks, on getting up one day, Nov. 11th, 1821, he felt weak and giddy, and was unable to stand. Red streaks were observed on the cheeks, and a red spot upon the ala nasi. The latter by the next day had spread all over the nose, and head-ache and fever came on. Before night, a livid vesicle, *(ein blauschwarzes Blätterchen,)* appeared upon the ala, where the red spot had been, and gradually increased. The nose, eyelids, and soon the whole face, were swollen, hard, of a dingy red, and shining; and on the third day, he was taken to the Hospital.

A number of livid vesicles of the size of peas, and with *very hard* bases, were now seen on the nose; the integuments around were much indurated, the tongue loaded with yellow fur, the pulse 76 and weak, the spirits dejected, deglutition difficult, and there was great thirst.

On the 4th day, the swelling was increased, the tip of the nose gangrenous, the upper lip beset with vesicles, and *an offensive corrosive discharge was taking place from both nostrils.* Blood taken from the arm presented a firm buffy coat.

On the 5th day, the whole nose and upper lip were gangrenous, the swelling was greater, and there were pustules on the forehead like those already described *(neue Puslein von derselben Beschaffenheit wie die schon früher beschriebenen.)*

On the 6th, more pustules arose on the forehead; the nose and upper lip were black, cold, and senseless.

On the 7th, red spots, passing quickly into suppuration, arose on various parts, especially on the fore-arms and legs. The nose was quite stopped up: the discharge from it mixed with blood, and so corrosive, as to destroy a portion of the slough of the upper lip. The breath stank.

On the 8th, the pustules throughout the body and extremities were more numerous and livid; the pulse was 140; nearly the whole face gangrenous; and in the evening he had a very offensive evacuation from the bowels, and soon afterwards expired.

Dr. Schilling seems satisfied that the disease arose from the contagion of a glandered horse, many of which wretched animals, kept for dissection and operations in the veterinary school, the patient had been looking after for some months, and had frequently, he said, washed out their nostrils.

Under the sternum in the seat of the thymus, and upon the tendinous portion of the left temporal muscle, a gelatinous yellow mass, similar to what is often met with in the cellular membrane of anasarcous persons, was found. 'The pericranium, especially of the frontal bone, was sown, as it were, with yellow pustules (tubercles?) of the size of millet seeds, and the bones below were sound.' 'The bones of the nose were decidedly carious, while the others of the face were healthy. Under every part of the skin where the earliest pustules took place, the cellular membrane was changed to a gelatinous mass, like that already mentioned. Pustules (tubercles?) were found even in the substance of the muscles, projecting, as it were, from their fibres, and containing a puriform lymph, but not clustered like those on the head. The fibres around were half liquified.'

As a postcript, the editor relates the following case, by Dr. Weiss, Kreis-Chirurgus at Neumarkt.

II. Gottfred Kliesch, aged 19, of delicate constitution, the son of poor parents, had always worked in various places, but latterly been employed in a farmer's stables at Bischdorf. According to his father's very imperfect account, he had frequently had eruptions, and frequently glandular swellings in his neck, many of which had suppurated. Whether any slight ulceration still existed on his face or neck, is not said. He had been vaccinated with success.

For several weeks he had complained, the father said, of pain in the head and back, weariness, and weakness of his limbs, loss of appetite, and disturbed sleep, all which he ascribed to lifting heavy sacks of corn; but continued at his occupation, a part of which consisted in looking after a *glandered horse*, which was with an ox in a separate stable. The stench, in cleaning out the manger, was such as continually to make him sick, and at last he took to his bed. He was sent to his parents Oct. 12th, that he might be taken better care of, and got worse daily till the 24th, when Dr. Weiss was requested to see him.

'I found him,' says this gentleman, 'in a miserable bed; although the room was filled with the smoke of juniper berries, there was a bad smell around the patient. He lay emaciated, in a mild delirium, his eyes dull and sunk, his nostrils somewhat extended, their inner lining, as far as the outer edge, covered with superficial ulcerations, some of which also were seen on the lips; the tongue and teeth were encrusted with yellow sordes. *From the mouth and nostrils there was an abundant yellow puriform discharge;* and all the glands beneath the lower jaw were swollen. All over the body, but especially on the lower extremities, were pustules, healed in some places, ulcerated in others and foul. The genitals were healthy, and the inguinal glands moderately enlarged. His strength was very prostrate; he answered me rationally, but in so weak a voice as scarcely to be understood; respiration was laborious and difficult; the breath very offensive; the abdomen rather tumid, but not tender. Black liquid blood, extremely offensive, was discharged involuntarily from his bowels; the skin was dry and hot; the pulse frequent and weak. He died the next day.'" 211.

Rust subjoins his conviction that both these patients died of the glanders caught from the diseased horses which they took care of.

In the 17th volume of the same work (1824) is the case of one Martin Tesmer, aged 25, who, from the 15th to the 27th July, 1823, suffered from pain in his limbs, side, and head, with thirst, loss of appetite, restlessness, and a pricking sensation at the tip of the nose. About the 29th inflammation of the left cheek, the nose, and upper lip, came on. There was considerable pyrexia. Aug. 1st. The inflammation had spread, and the extremity of the nose appeared to be suppurating. On careful enquiry, the lad acknowledged that he had had the care of two diseased horses, one of whom was affected with great discharge from the nostrils. This discharge he had been sprinkled with while giving the horse some medicine.

"On the 3d, the end of the nose was covered with a hard, painful, gangrenous scab; several pustules as large as peas and filled with yellowish fluid (phlyzaceous), arose around it, and on the upper lip: the whole face was swollen, and the inside of the nose inflamed. On the 4th, the entire face, and inside of the mouth, were inflamed and swollen, nearly half the nose destroyed, and the rest covered by a gangrenous scab. There were more pustules on the cheeks, some discharging an acrid ichor: the fever was much more intense. Blood taken from the arm on the 5th day, showed a strong inflammatory buff. On the 6th day, the swelling and destruction of the face proceeded, and even the gums and tongue were implicated: pustules, like those of the face, arose on the chest and extremities.

On the 7th, *a copious stinking ichor proceeded from the nose and mouth;* the gangrene extended; more pustules appeared upon the forehead, the angle of the lower jaw, and the body. The pulse was 130, and small; there was delirium, and colliquative sweats.

On the 8th, the symptoms were all greatly increased; the pulse could not be counted, and death took place. Previously and subsequently to his decease, the stench was excessive; and on this account the body could not be inspected. The horse was examined, and found to have a swelling in the glands of the neck, with a copious mucous and purulent discharge from the nose. When killed, the right lung was full of pus, and the left of abscesses and *tubercles,* although no disease was visible in the mucous membranes of the nostrils." 213.

In the second part of the 14th volume, is an account, by Dr. Tarozzi, of Ostiano, of eleven persons affected, in 1818, in a similar manner, and of whom all but one died, after visiting, with about 35 others, an ill-ventilated stable, 20 feet square, containing three cows and ten horses, one of which had laboured under an offensive discharge from the nostrils for a twelvemonth, and none of which had left the stables for three months.

"Violent pyrexia, pains, spasms, boils, and at last a large carbuncle, generally characterised the first stage; gangrenous vesicles and typhus were the chief features of the second. These were not the precise symptoms observed in the preceding cases; and as the parish priest, who closely attended on all the patients, also caught the disease, and as a discharge from the nostrils is not stated to have taken place in any one instance, and the accounts are in many respects defective, I dare not give an opinion that this disease, any more than that of the proprietor of the horse which infected Tesmer, was really glanders. It might have been an affection similar to a carbuncular epidemic which seemed to be derived from brutes,—from skinning sheep and oxen that had died of *blutseuche* or *milz-brand,* at Merseburg, in 1822, and described in the first part of the same volume; to the cases of butchers and others affected with gangrenous erysipelas, pustules, carbuncle, &c. apparently from contact, even without abrasion, with the blood of diseased oxen, related by M. Morand; to the disease described by MM. Enaux and Chaussier, under the title of *Pustule Maligne;* and to a disease which proved fatal to two men whose hands, though sound, and washed four minutes afterwards, were wetted, in the performance of venesection, with the blood of a cow labouring under *milz-brand,* and in whom the chief local affection was peritoneal inflammation and effusion." 215.

We have thus given a long account of the longest paper in the recent volume of the Medico-Chirurgical Transactions; and placed all the facts fairly before our readers. We could add, from our own experience, some curious particulars respecting diseases that have been caught from domestic animals; but this article has extended to such a length that we are compelled to bring it to a close.

